# Supplementary material for: New Coelomycetous Fungi from Freshwater in Spain
Source: J Fungi (Basel). 2021 May 8;7(5):368. doi: 10.3390/jof7050368 (PMC8151841; doi:10.3390/jof7050368)
Supplement: Supplementary file 1 [file jof-07-00368-s001.zip › Figure S3.pdf]

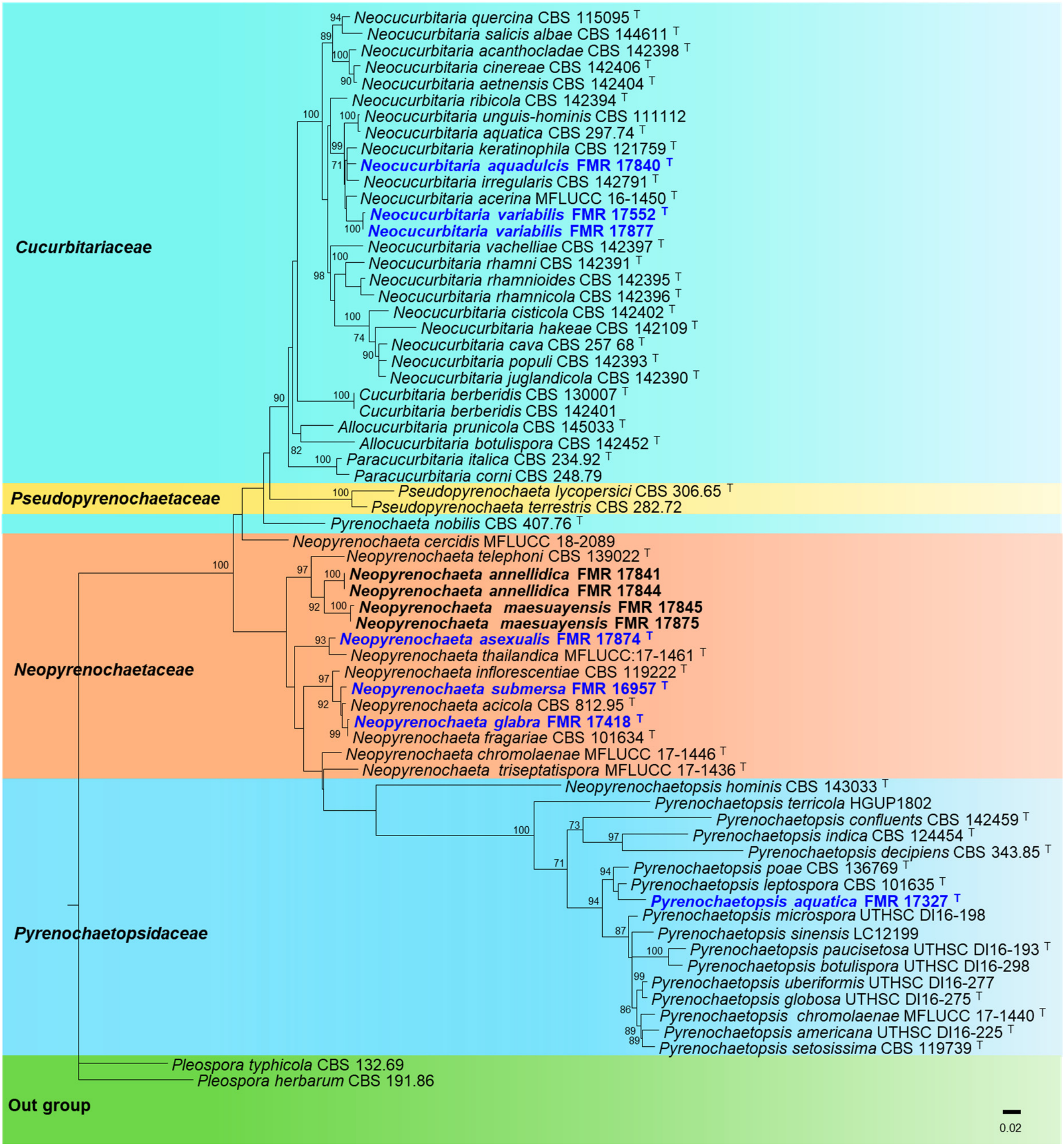

Figure S3. ML phylogenetic tree of *Cucurbitariaceae*, *Neopyrenochaetaceae*, *Pseudopyrenochaetaceae*, and *Pyrenochaetopsidaceae* inferred from *rpb*2 sequences (734 bp). Support in nodes is indicated above branches by bootstrap values of 70 % or higher. T = ex-type strains. New species are indicated in **blue**. New strains isolated during this study are indicated in **bold**.
