# Supplementary material for: New Coelomycetous Fungi from Freshwater in Spain
Source: J Fungi (Basel). 2021 May 8;7(5):368. doi: 10.3390/jof7050368 (PMC8151841; doi:10.3390/jof7050368)
Supplement: Supplementary file 1 [file jof-07-00368-s001.zip › Table S1_new.pdf]

Supplementary Table S1. Fungal taxa sequences used in this study.

**Taxon**	**Strain**	**GenBank Accession Number**			
		**LSU**	**ITS**	***tub2***	***rpb2***
Allocucurbitaria botulispora	CBS 234.92 [T]	LN907416	LT592932	LT593001	LT593070
Allocucurbitaria prunicola	**CBS 145033 [T]**	**MK442534**	**NR_166273**	**MK442737**	**MK442668**
Cucurbitaria berberidis	CBS 142401	MF795756	MF795756	MF795886	MF795798
Cucurbitaria berberidis	CBS 130007 [T]	KC506793	LT717673	LT717676	LT854936
Neocucurbitaria acanthocladae	CBS 142398 [T]	MF795766	NR_156354	MF795894	MF795808
Neocucurbitaria acerina	MFLUCC 16-1450 [T]	NG_059784	NR_154254	---	---
Neocucurbitaria aetnensis	CBS 142404 [T]	MF795769	NR_156355	MF795897	MF795811
Neocucurbitaria aquatica	CBS 297.74 [T]	EU754177	LT623221	LT623238	LT623278
Neocucurbitaria cava	CBS 257.68 [T]	EU754199	JF740260	KT389844	LT717681
Neocucurbitaria cinereae	CBS 142406 [T]	MF795771	NR_156356	MF795899	MF795813
Neocucurbitaria cisticola	CBS 142402 [T]	MF795772	NR_156357	MF795900	MF795814
Neocucurbitaria aquadulcis	**FMR 17840 [T]**	**LR897771**	**LR897770**	**LR897794**	**LR897793**
Neocucurbitaria hakeae	CBS 142109 [T]	KY173526	KY173436	KY173613	KY173593
Neocucurbitaria irregularis	CBS 142791 [T]	LN907372	LT592916	LT592985	LT593054
Neocucurbitaria juglandicola	CBS 142390 [T]	MF795773	NR_156358	MF795901	MF795815
Neocucurbitaria keratinophila	CBS 121759 [T]	LT623215	EU885415	LT623236	LT623275

Neocucurbitaria populi	CBS 142393 T	MF795902	NR_156359	MF795902	MF795816
Neocucurbitaria prunicola	CBS:145033 T	MK442534	MK442594	---	MK442668
Neocucurbitaria quercina	CBS 115095 T	GQ387619	LT623220	LT623237	LT623277
Neocucurbitaria rhamni	CBS 142391 T	MF795775	---	---	MF795817
Neocucurbitaria rhamnicola	CBS 14239 6T	MF795780	NR_156360	MF795906	MF795822
Neocucurbitaria rhamnioides	CBS 142395 T	MF795782	NR_156361	MF795908	MF795824
Neocucurbitaria ribicola	CBS 142394 T	MF795785	NR_156362	MF795911	MF795827
Neocucurbitaria salicis albae	CBS 144611 T	MK442535	NR_16336	MK442738	MK442669
Neocucurbitaria unguis hominis	CBS 111112	GQ387623	LT623222	LT623239	LT623279
Neocucurbitaria vachelliae	CBS 142397 T	MF795787	NR_156363	MF795913	MF795829
Neocucurbitaria variabilis	**FMR 17877**	**LR897785**	**LR897784**	**LR897807**	**LR897806**
Neocucurbitaria variabilis	**FMR 17552 T**	**LR897769**	**LR897768**	**LR897792**	**LR897791**
Neopyrenochaeta acicola	CBS 812.95 T	GQ387602	LT623218	LT623232	LT623271
Neopyrenochaeta annellidica	**FMR 17841**	**LR897773**	**LR897772**	**LR897796**	**LR897795**
Neopyrenochaeta annellidica	**FMR 17844**	**LR897775**	**LR897774**	**LR897798**	**LR897797**
Neopyrenochaeta annellidica	MFLU 11-1105 T	MT183502	MT185538	---	---
Neopyrenochaeta cercidis	MFLU 18-2089	MK347932	MK347718	---	MK434908
Neopyrenochaeta chiangraiensis	MFLUCC 17-1445 T	MT214468	NR_168875	---	---
Neopyrenochaeta chromolaenae	MFLUCC 17-1446 T	MT214469	NR_168876	---	MT235824

Neopyrenochaeta submersa	**FMR 16957** T	**LR897765**	**LR897764**	**LR897787**	**LR897786**
Neopyrenochaeta glabra	**FMR 17418** T	**LR897767**	**LR897766**	**LR897790**	**LR897789**
Neopyrenochaeta neothailandica	**FMR 17874** T	**LR897779**	**LR897778**	**LR897802**	**LR897801**
Neopyrenochaeta fragariae	CBS 101634 T	GQ387603	LT623217	LT623231	LT623270
Neopyrenochaeta inflorescentiae	CBS 119222 T	EU552153	EU552153	LT623233	LT623272
Neopyrenochaeta maesuayensis	**FMR 17845**	**LR897777**	**LR897776**	**LR897800**	**LR897799**
Neopyrenochaeta maesuayensis	**FMR 17875**	**LR897781**	**LR897780**	**LR897804**	**LR897803**
Neopyrenochaeta maesuayensis	**FMR17876**	**LR897783**	**LR897782**	**---**	**LR897805**
Neopyrenochaeta maesuayensis	MFLU:15-0078	MT183504	MT185540	---	---
Neopyrenochaeta telephoni	CBS 139022 T	KM516290	KM516291	LT717678	LT717685
Neopyrenochaeta thailandica	MFLUCC 17-1461 T	NG_068716	MT214376	---	MT235825
Neopyrenochaeta triseptatispora	MFLUCC 17-1436 T	MT214471	MT214377	---	MT235826
Neopyrenochaetopsis hominis	CBS 143033 T	LN907381	LT592923	LT592992	LT593061
Paracucurbitaria corni	CBS 248.79	GQ387608	LT903672	LT900365	LT903673
Paracucurbitaria italica	CBS 234.92 T	EU754176	LT623219	LT623235	LT623274
Pleospora herbarum	CBS 191.86 T	JX681120	NR_111243	---	KC584471
Pleospora typhicola	CBS 132.69	JF740325	---	KT389843	KC584505
Pseudopyrenochaeta lycopersici	CBS 306.65 T	EU754205	NR_103581	LT717674	LT717680
Pseudopyrenochaeta terrestris	CBS 282.72 T	LT623216	LT623228	LT623246	LT623287

Pyrenochaeta nobilis	CBS 407.76 [T]	EU754206	EU930011	KT389845	LT623276
Pyrenochaetopsis americana	UTHSC:DI16-225 [T]	LN907368	LT592912	LT592981	LT593050
Pyrenochaetopsis botulispora	UTHSC:DI16-298	LN907432	LT592941	LT593010	LT593080
Pyrenochaetopsis chromolaenae	MFLUCC 17-1446 [T]	MT214469	NR_168876	---	MT235824
Pyrenochaetopsis confluents	CBS 142459 [T]	LN907446	LT592950	LT593019	LT593089
Pyrenochaetopsis decipiens	CBS 343.85 [T]	GQ387624	LT623223	LT623240	LT623280
Pyrenochaetopsis aquatica	**FMR 17327 [T]**	**LR216649**	**LR216648**	**LR897788**	**LR216647**
Pyrenochaetopsis globosa	UTHSC:DI16-275 [T]	LN907418	LT592934	LT593003	LT593072
Pyrenochaetopsis indica	CBS 124454 [T]	GQ387626	LT623224	LT623241	LT623281
Pyrenochaetopsis leptospora	CBS 101635 [T]	GQ387627	JF740262	LT623242	LT623282
Pyrenochaetopsis microspora	UTHSC:DI16-198	LN907341	LT592899	LT592968	LT593037
Pyrenochaetopsis paucisetosa	UTHSC:DI16-193 [T]	LN907336	LT592897	LT592966	LT593035
Pyrenochaetopsis poae	CBS 136769 [T]	KJ869175	KJ869117	KJ869243	LT623286
Pyrenochaetopsis setosissima	CBS 119739 [T]	GQ387632	LT623227	LT623245	LT623285
Pyrenochaetopsis sinensis	LC12199	MK348581	MK348586	MK348221	MK355077
Pyrenochaetopsis tabarestanensis	CBS 139506	KF803343	KF730241	KX789523	---
Pyrenochaetopsis terricola	HGUP1802	MH697393	MH697394	MH697392	MH697395
Pyrenochaetopsis uberiformis	UTHSC:DI16-277 [T]	LN907420	LT592935	LT593004	LT593074

[1]**CBS**: Culture collection of the Westerdijk Biodiversity Institute, Utrech, The Netherlands; **FMR**: Facultat de Medicina, Reus, Spain; **HGUP**: Corresponding author's personal collection deposited in laboratory, housed at Guizhou, China; **LC**: Corresponding author's personal collection deposited in laboratory, housed at CAS, China; **MFLU** : Mae Fah Luang University Herbarum, Chiang Rai, Thailand; **MFLUCC** : Mae Fah Luang University Culture Collection, Chiang Rai, Thailand ; UTHSC, Fungus Testing Laboratory at the University of Texas Health Science Center, San Antonio, Texas, USA. [2]Strains studied by us are indicated in **bold**. [3]ITS: internal transcribed spacer region 1 and 2 including 5.8S nrDNA; LSU: large subunit of the nrRNA gene; *rpb2*: RNA polymerase II second subunit; *tub2*: ß-tubulin. [T]Ex-type strain.
