# Supplementary material for: New Coelomycetous Fungi from Freshwater in Spain
Source: J Fungi (Basel). 2021 May 8;7(5):368. doi: 10.3390/jof7050368 (PMC8151841; doi:10.3390/jof7050368)
Supplement: Supplementary file 1 [file jof-07-00368-s001.zip › Table S2_new.pdf]

Supplementary Table S2. Closest hits to our strains (FMR) of interest after a blast search of NCBI's GenBank nucleotide database.

**Accession number**	**ITS / identity**	**LSU / identity**	***tub*2 / identity**	***rpb*2 / identity**
FMR 17552	*Neocucurbitaria unguis-hominis* CNRMA 4.1112 / 96 %	*Neocucurbitaria vachelliae* CBS 142397 / 99 %	*Neocucurbitaria acerina* CBS 142397 / 94 %	*Neocucurbitaria acerina* CBS 142403 / 95 %
FMR 17840	*Neocucurbitaria unguis-hominis* CBS 111112 / 97 %	*Neocucurbitaria salis-albae* CBS 144611 / 99 %<br>*Neocucurbitaria keratinophila* CBS 121759 / 99 %<br>*Neocucurbitaria quercina* CBS 297.74 / 99 %	*Neocucurbitaria keratinophila* CNM-CM 8674 / 94 %	*Neocucurbitaria acerina* CBS 142403 / 96 %<br>*Neocucurbitaria keratinophila* CNM-CN8674 / 96 %
FMR 16957	*Neopyrenochaeta acicola* MUT<ITA>:4382 / 100 %	*Neopyrenochaeta acicola* MUT<ITA>:4382 / 100 %	*Neopyrenochaeta acicola* CBS 101634 / 98 %	*Neocucurbitaria acicola* CBS 812.95 / 98 %
FMR 17418	*Neopyrenochaeta acicola* CBS 101634 / 100 %	*Neopyrenochaeta acicola* MUT<ITA>:4382 / 100 %	*Neopyrenochaeta acicola* CBS 101634 / 98 %	*Neopyrenochaeta acicola* CBS 101634 / 99%
FMR 17874	*Neopyrenochaeta thailandica* MFLUCC 17-1461 / 98 %	*Neopyrenochaeta thailandica* MFLUCC 17-1461 / 100 %	*Neopyrenochaeta acicola* CBS 101634 / 92 %	*Neocucurbitaria thailandica* MFLUCC 17-1461 / 96 %
FMR 17327	Pyrenochaetopsis leptospora P6589* / 100 %	*Pyrenochaetopsis leptospora* CBS 101635 / 99 %	*Pyrenochaetopsis leptospora* CBS 101635 / 95 %	*Pyrenochaetopsis leptospora* CBS 122787 / 95 %

CBS: Westerdijk Fungal Biodiversity Institute, Utrecht, The Netherlands; ; CNM-CM: National Centre for Microbiology, Instituto Carlos III, Madrid, Spain; CNRMA: National Reference Center for Invasive Mycoses and Antifungals; Institut Pasteur, Paris, France; FMR: Faculty of Medicine culture collection, Reus, Spain; MUT: Mycotheca Universitatis Taurinensis, Turin, Italy; MFLUCC: Mae Fah Luang University Culture Collection, Chiang Rai, Thailand. * Sequence deposited by J. G. Macia-Vicente at NCBI databases.
